# A Radio-Aware Routing Algorithm for Reliable Directed Diffusion in Lossy Wireless Sensor Networks

**DOI:** 10.3390/s91008047

**Published:** 2009-10-13

**Authors:** Yong-Pyo Kim, Euihyun Jung, Yong-Jin Park

**Affiliations:** 1 Department of Electronics and Computer Engineering, Hanyang University/Haengdang-dong, Sungdong-gu, Seoul, 133-791, Korea; E-Mails: ypkim@hyuee.hanyang.ac.kr (Y.-P.K.); park@hyuee.hanyang.ac.kr (Y.-J.P.); 2 Department of Computer Science, Anyang University/Samseong-ri, Buleun-myeon, Ganghwagun, Inchon, 417-833, Korea

**Keywords:** WSNs, lossy WSNs, radio-aware, directed diffusion, routing algorithm, reliability, cross-layer

## Abstract

In Wireless Sensor Networks (WSNs), transmission errors occur frequently due to node failure, battery discharge, contention or interference by objects. Although Directed Diffusion has been considered as a prominent data-centric routing algorithm, it has some weaknesses due to unexpected network errors. In order to address these problems, we proposed a radio-aware routing algorithm to improve the reliability of Directed Diffusion in lossy WSNs. The proposed algorithm is aware of the network status based on the radio information from MAC and PHY layers using a cross-layer design. The cross-layer design can be used to get detailed information about current status of wireless network such as a link quality or transmission errors of communication links. The radio information indicating variant network conditions and link quality was used to determine an alternative route that provides reliable data transmission under lossy WSNs. According to the simulation result, the radio-aware reliable routing algorithm showed better performance in both grid and random topologies with various error rates. The proposed solution suggested the possibility of providing a reliable transmission method for QoS requests in lossy WSNs based on the radio-awareness. The energy and mobility issues will be addressed in the future work.

## 1. Introduction

Wireless Sensor Networks (WSNs) are an emerging technology that has gained great interest from researchers due to the widespread availability of cheap and tiny sensors. WSNs can be defined as a collection of many tiny sensor nodes forming a self-structured network interconnected by a wireless technology. Since sensor nodes suffer from restricted computing resources and battery life time, the development of effective routing protocols for WSNs have been heavily debated after WSNs were introduced [1,2].

In the view of limited energy and computing resources, routing protocols for WSNs should be differently designed compared to routing protocols for other wireless networks (i.e., MANETs) which mainly focus on data transmission speed or network throughput [3,4]. In order to address the factors restricting WSNs routing, many routing protocols have been proposed [5]. In [5], authors summarized diverse routing strategies which consider several issues for WSNs routing: in-network processing [6,7], clustering [8–10], and data-centric methods [11,12].

These earlier research studies contributed to diverse domains such as energy efficiency, abstract data access, real time data transmission, etc. Although the diverse approaches have shown their own initiatives in this research area, most studies have the common assumption that routing protocols are run in an ideal network environment without errors. In other words, existing studies did not consider data losses and route failure of the wireless networks due to unpredictable errors such as node failure, contention, interference or fading [13,14].

Directed Diffusion (DD) [12] is a prominent WSNs routing protocol supporting data-centric routing, but it also assumes an ideal network environment. In DD, a flood of interests representing application requests plays an important role. During interest propagation period for a route setup, the routing information, gradient, is calculated and stored in each node's routing table while an interest floods. After the interest has been flooded, a sensor node with matching sensing data is triggered to deliver the data to a sink node by using its gradients via the intermediate nodes. The problem is that DD is poor at dealing with the network errors after the gradient is calculated once. In DD, if the lossy situation occurs during a data transmission period, the node cannot send data any more to the given path until a new route setup period is restarted. Consequently, control packets should be flooded into the whole network in order to resolve the lossy situation problem, but frequent flooding of control packets causes a sensor node to consume its energy rapidly. Therefore, when WSNs are exposed to a lossy network environment, DD will suffer from low data delivery rates and excessive energy consumption from frequent flooding of control packets. In order to resolve this issue, a more robust routing algorithm which can handle the lossy wireless environment should be considered. The routing algorithm should be resistant to network errors without additional processing and can decide a routing path by capturing the current status of the WSNs.

Recently, a concept of cross-layer design has emerged to utilize radio information which reflects the current status of the wireless network. The radio information is considered as an important design factor to provide reliable data transmission services with WSNs [15,16]. The cross-layer design is a mechanism to enhance the performance of wireless communication at higher layers by utilizing information of lower layers like PHY and MAC layers. There are many researches to use the cross-layer design, but they mainly focused on the cooperation with lower layers (e.g., PHY and MAC) [17,18]. We expected the improvement of the network performance by using the cross-layer design at the network layer on DD.

In this paper, we proposed a Radio-Aware (RA) routing algorithm which can improve the reliability of DD in lossy WSNs. The proposed algorithm can provide an optimum route to react to lossy WSNs based on the radio information which reflects the current network status by using a cross-layer design. Furthermore, if RA detects the lossy situation at a target node, it can choose an alternative neighbor node as a next hop to provide reliable data transmission. Since we added the cross-layer design that utilizes radio information of existing packets, RA has the advantages of reliable communication and interoperability in lossy WSNs without burdening additional overhead on DD. In order to evaluate RA, several simulations were performed with *ns*-2 under various conditions. In accordance with the simulation results, the proposed algorithm showed better performance in various environments with different error rates.

The remainder of the paper is organized as follows. In Section 2, we discuss related works and motivation. Section 3 describes the proposed radio-aware routing algorithm. In Section 4, we explain the evaluation metrics of the simulation. The simulation results are analyzed in Section 5. Section 6 concludes the paper and addresses future work.

## 2. Related Works and Motivation

### 2.1. The Overview of Directed Diffusion

DD [12] is a data-centric routing protocol based on the distributed mode. In DD, the propagation and aggregation of both interest and sensing data is determined by localized message exchanges between neighbor nodes. The next hop was only decided as a next route for multi-hop communication instead of whole route path. The operation of DD is divided into the two periods of interest propagation and the data aggregation.

In the first period, a sink and sensor nodes collect the routing information by propagating an interest for transmitting their data. When a sensor application requests particular sensing data, a sink creates an interest specifying the target data and then disseminates the interest over WSNs. The interest represents the application's request including attribute-value pairs that indicate target sensing data. When the interest arrives at each node, the node checks whether the request matches its own sensing data or not. At the same time, the node sets up a gradient toward the direction to the sink node and forwards the interest to its neighbors. After setting up gradients, the route can be determined at each node with its value of gradient using the routing algorithm of DD.

**Figure 1.**
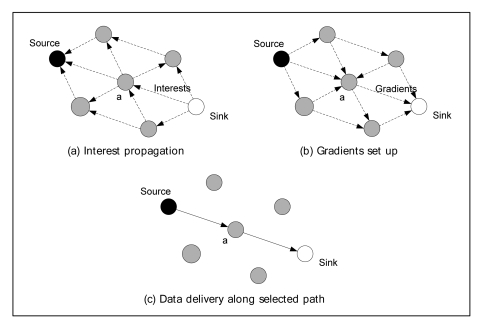
The communication flow of Directed Diffusion.

The second period is started after interests are propagated across the network. In the second period, sensors are triggered to start sending the data and a sensor application begins collecting data. The target sensing data are aggregated to a sink based on the route with the value of gradients. Figure 1(a) shows the process of interest flooding into the whole network. Figure 1(b) shows the step for setting up the gradient at each node through interest propagation period from the sink node to a source node. The data transmission state along the selected route is shown in Figure 1(b).

There are several versions of DD. The original, Two Phase Pull (TPP) offers efficient communication between sources and sinks. TPP consists of two phases of interactions which can provide data collection service using several control packets: interest, exploratory data, and positive/negative reinforcement.

The first phase of TPP is composed of the interest flooding and exploratory data to find source nodes and to set up gradient at all nodes. The initial data from the source node is marked as exploratory data and is sent again to all neighbors which has matching gradients. The second phase of TPP is a step to reinforce the route and to send data through the reinforced route. The positive and negative reinforcement packets are used to increase or decrease the gradient on a certain route. Especially, the negative reinforcement suppresses loops or duplicated routes that may arise due to the network changes. After the route was reinforced, sensing data is sent through the reinforced route to a sink.

The control packets of TPP for recovering network changes enable TPP to provide robust communication in WSNs. However, since too many interest and exploratory packets flooded to the networks, the energy of each sensor node tended to be depleted rapidly in TPP.

In order to overcome the problems of TPP, One Phase Pull (OPP) was proposed to eliminate explicit exploratory data and reinforcement packets. OPP has only one phase to provide collecting sensing data using an interest packet only compared to TPP. OPP used the interest only to set up the gradient and sent the data directly on the selected route without additional control packets. This simple feature of OPP is a significant benefit because it can minimize the flooding of TPP. When an interest arrives at a source node, it does not mark its first data as exploratory data. The source node sends data only on the route with preferred gradient. The preferred gradient is determined by the neighbor who was the first to send the matching interest. Therefore, in OPP, the lowest latency route is implicitly reinforced without requiring additional reinforcement packets.

OPP contributed to saving energy at each node which results in extending the network life time without requiring additional control packets of exploratory data and reinforcement packets. However, the communication route between sources and a sink is assumed to be asymmetric due to the different direction when the route is determined during interest propagation period. The novel method should be considered to overcome the link asymmetry problem at the protocol level.

### 2.2. The Motivation

Although DD was proven to have several advantages such as providing a robust, scalable and energy efficient routing scheme, it has several limitations. Firstly, the protocol overhead should be reduced to minimize flooding of control packets. Although the control packets are essential to set up a route from route failure or data loss, increasing flooding packets causes excessive energy depletion of sensor nodes. Secondly, other considerable metrics for route decision had to be applied to support various QoS such as data rate, energy, reliability, or latency. However, there is little research to change the gradient to other metrics as well as DD. That is, the data rate is still the only metric used to set up gradients in DD.

Thirdly, DD cannot deal with lossy WSNs resulted from the unstable network, because a single session of DD is separated into interest propagation and data aggregation periods. If the lossy situation occurs in the data aggregation period, a node cannot send sensing data along the pre-decided route because DD cannot make a new decision to recover the lossy situation on the route in the data propagation period promptly.

Finally, link asymmetry may be a considerable issue in wireless networks. A supplement is needed to remedy the asymmetry of a routing protocol, because the data path between nodes and a sink is determined by interest propagation from a sink to sensor nodes in DD. A routing protocol for lossy WSNs has to address the asymmetry characteristics to provide link symmetry.

In order to resolve these issues of DD, two kinds of approaches has been introduced. Firstly, some studies focus on prolonging the network life time using energy awareness. In [19,20], the authors used the concepts of clustering to extend the network's life time. These concepts contributed to reducing the energy consumption by sending interest between the cluster heads instead of interest flooding to the whole networks. In [21], the maximum gradient depth and minimum remaining energy were used to select the next hop. The average network lifetime was extended and the load balance was also improved in this scheme. Secondly, there are research supporting QoS that provide different data transmission strategies by considering different types of data traffic such as real time traffic and ordinary data traffic. In [22], the authors pointed out that DD cannot support time sensitive data traffic and a load-balanced routing scheme. They designed a Real Time (RT) filter and a Best Effort (BE) filter to support corresponding QoS. In this approach, the RT filter showed the shorter end-to-end delay and the BE filter achieved better energy efficiency.

Generally, real WSNs should be assumed as unstable due to the nature of wireless communication. The instability of WSNs can be defined as lossy WSNs including many reasons: contention, interference, node mobility, fading by object, or node failure. Lossy WSNs are our error model to emulate real sensor networks having those unpredictable errors. The unpredictable data losses and route failures may occur in lossy WSNs. This random failure can be also defined as the lossy situation in our assumption.

The earlier studies tried to overcome the problems of DD and contributed to extending network lifetimes by considering energy, load balancing, or QoS. However, the previous studies cannot provide the reliable data transmission in lossy WSNs, because they assumed that the network is stable. Furthermore, the previous studies are lack of methods to complement link asymmetry of the wireless networks. In the previous studies, they carry over the supplement of link symmetry to the detecting and filtering function of MAC layer [12].

When the network topology becomes unstable, the lossy situation of DD will increase and DD needs to flood more control packets across the network to adjust the error conditions. Essentially, since DD is separated into the route setup (interest propagation period) and data transmission (data aggregation period), DD cannot send sensing data along existing routes when the WSNs become unstable. The more data loss control packets are flooded, the lossier situation occurs due to the rerouting, retransmission, and route failure.

In order to overcome the lossy situation, a routing algorithm is required to consider up-to-date network conditions. If the routing algorithm can recognize the current status of WSNs, the lossy situation can be adjusted to provide a reliable data collecting service to sensor applications under lossy WSNs.

## 3. Proposed Routing Algorithm

The proposed algorithm (RA) is different from previous works that try to improve DD by using the additional control session. The novelty of the proposed routing algorithm is that RA is designed to remove the burden of additional control session. By adopting cross-layer design instead of the additional control session, RA can deal with the lossy situation without additional overhead.

The previous algorithms need an additional control session to deal with lossy situation within a transport layer which controls the error recovery process such as the retransmission of control packets, rerouting, and error correction process. The additional control session can recover the lossy situation, but it imposes an additional overhead and complexity on the original features. Furthermore, their modification of DD without consideration of the radio information may give disadvantages to break originality and to burden overhead and complexity.

DD already has the traditional control session at the application layer [23] and this functionality is used to ease the failed route problem. This functionality of DD operates well in most lossy situation, but DD suffers severely from communication overhead due to the flooding packets of the additional control session. It is obvious that the additional control session results in degradation of the communication throughput and transmission delay.

Furthermore, as the authors of [24] pointed out, the inability of the previous algorithms of wired networks is not suitable for wireless networks to correctly identify the cause of packet losses. It is important to know the details of current status of wireless network, but the early works did not consider the radio information which can identify the cause of errors reflecting current status in lossy WSNs. In the previous works, since the network layer has no method to know the details of the current network condition, the packet should be transmitted from low layers to a network layer so that the routing algorithm is able to utilize the radio information.

On the other hand, RA can provide the method to utilize the radio information across the layers between the MAC and the network layers using cross-layer design. RA updates the radio information into the neighbor table. The neighbor table is shared between MAC and network layer to decide a route with best gradient. When the network becomes unstable in lossy WSNs, RA also suffers from the lossy situation. However, RA is able to restore the route in according to the variant network status. RA can provide reliable data collecting service without additional overhead and complexity for optimizing the route in lossy WSNs.

### 3.1. Consideration of 802.11 MAC for Sensor Network

Several MAC protocols (SMAC, 802.15.4, etc) can be candidates for WSNs, because the MAC protocols of WSNs should minimize energy consumption. However, as various sensor applications are developed (i.e., multimedia sensor network), WSNs have to deal with new requirements such as scalability, throughput, QoS (reliability, security, low latency, etc), and interoperability with existing infrastructure. Although a specific sensor MAC gives several advantages of saving more energy on its operation, the restricted energy efficient MAC cannot support all of the various requirements.

In [25], they showed the advantages of 802.11 sensor networks as follows. Firstly, since 802.11 networks are widely deployed and deployments continue at a rapid rate for multiple applications, the distribution of sensor networks with existing infrastructure can be naturally easy. Secondly, the information of sensed data can be easily prioritized and transmitted with the higher utilization of the 802.11 network such as QoS, multi-rate, and cross-layer design. Thirdly, the scalability to utilize higher data rate sensors is supported up by 802.11 family protocols. 802.11 can scale to a greater number of nodes and faster data rates with various QoS.

The author of DD also used a modified 802.11 MAC to emulate realistic sensor network radio more closely. They altered the ns-2 radio model to fit a sensor radio specification minimizing energy consumption. The energy efficiency of 802.11 MAC has to be considered, but we believe the effect of the various utilizations of 802.11 MAC to give more advantages to WSNs.

### 3.2. Operation of the Radio-Aware Routing Algorithm

In this paper, we proposed a radio-aware (RA) routing algorithm to address lossy situation. RA not only maintains the advantages of DD, but also applies radio information to set up the route with cross-layer designs. In order to address the problem mentioned above, RA is designed to improve the reliability of DD in lossy WSNs. Firstly, RA can exchange routing information among nodes independent of the period. In RA, the route will be adjusted to the situation caused by unexpected errors in lossy WSNs.

Secondly, RA introduces radio information which represents the air time cost and route failure as new metrics of gradient. Since these new metrics can reflect current status of networks, RA can maintain consistent connectivity and achieve reliability in lossy WSNs. In addition to the metrics, the position of each sensor node is used to decide the closest direction from source nodes to a sink.

Finally, the fast re-route scheme is adopted to address the lossy situation in addition to the link asymmetry. In the fast re-route scheme, the lossy situation can be fetched through the RTS/CTS mechanism of the CSMA/CA at the 802.11b MAC. The route failure flag indicates the lossy situation of the error-prone node. RA can choose an alternative node instead of a failed node. Moreover, the fast re-route scheme gives reduction of link asymmetry problem of wireless network.

In RA, the air time cost and the position of each sensor node were collected to set up gradients whenever interest and data packets were received on each node. RA can calculate the air time cost, and the position of each node is included in the interest. If a source node has a problem in sending data due to route failure, RA can select an alternative route instead of failed one by utilizing fast re-reroute scheme.

#### 3.2.1. The air time cost

The air time cost (*C_a_*) represents the cost for occupying the channel when transmitting a single frame on the wireless channel. A route with the smaller air time cost can be considered as a better route than others, the route can be selected with higher priority for data routing. The air time cost can be measured from Equation (1):

(1) $$Ca=\left[O+\frac{Bt}{r}\right]\frac{1}{1-ef}$$

Var.Description*O*Overhead (699 μs)*Bt*Number of bits in test frame (8,192 bits)*r*Bit rate in Mbps*e_f_*Frame error rate

From Equation (1), as the frame error rate increases, the air time cost also increases according to the frame error rate. The air time cost (*C_a_*) was used in an 802.11b wireless mesh network [26] and it was redefined as a link quality in this paper. In Equation (1) *O* is the sum of *O_ca_* and *O_p_. O_ca_* is the channel access overhead and *O_p_* is the protocol overhead. *B_t_* is the number of bits in the test frame. Some representative values for these constants are: *O_ca_* = 335 μs, *O_p_* = 364 μs, and *B_t_* = 8,192 bits in 802.11b network. The input parameters of *r* and *e_f_* are the bit rate in Mbps and the frame error rate for the test frame size *B_t_*, respectively.

#### 3.2.2. The neighbor table

The radio-aware routing algorithm consists of three parts: gathering the radio information, route selection, and a fast-reroute scheme. In the first step, the radio information for the wireless network was collected and updated through the cross-layer design [27] at the MAC layer. The radio information indicates the link quality and route failure information which can be updated whenever receiving the interest and data packets from neighbors. Each node has a neighbor table to store the values of routing information. The table was designed to share information between MAC and network layers for cross-layer design.

When a node receives an interest packet from its neighbor, the node puts all the values of the neighbor node into the table, except the position of a sink. In our assumption, all the nodes are aware of their own position and the sink node's position when the nodes are deployed. However, since a node cannot know the neighbors' position, they have to exchange their position data. In order to exchange the position data, we added the data field to the interest packet. When an interest packet was propagated to neighbor nodes, each node put their position data into the interest instead of previous forwarding node's position data. If a node receives the interest from the neighbors, the node updates its neighbor table with the information of previous node.

The radio information is updated whenever a node receives both interest and data packets from neighbors to indicate the air time cost and a route failure flag. The air time cost can be calculated from Equation (1). The route failure flag is updated from 0 to 1 when a node fails to transmit sensing data to a target node for making a re-route decision.

The complete neighbor table was composed of a node ID, the position (x, y) of a node, the air time cost, and route failure flag as shown in Table 1. The Table 1 is a snapshot of node 33 at a simulation. In this case, node 23 would be selected as a next hop at the node 33, because node 23 has the smallest air time cost and the closest distance and the route failure flag is unset.

#### 3.2.3. The node selection algorithm

RA is originally designed to select an optimum route based on the distance and the air time cost. These two metrics of RA have an important role to make a route decision respectively. If a routing protocol decides a route based on the distance, the protocol can choose the shortest route between a source and a sink. However, the routing protocol only based on the distance information cannot properly cope with the lossy situation because the distance information does not have the information of the current network's status.

Whereas, if a routing protocol uses the air time cost for deciding the route, the protocol can choose the best route based on the current network's status. However, if a routing protocol only considers the air time cost, the direction of the data transmission can be opposite to the data forwarding route from a source to sink. The effect of inverse directed data transmission causes the long route or loop on the communicating route. For these reasons, we designed RA to take the advantages of each scheme for choosing optimum route based on two metrics to overcome lossy situation. The detailed operation of RA is as follows.

The air time cost was calculated and updated whenever receiving the interest and data from its neighbors. Also, the distance between each node and its sink node was calculated by examining the interest packet including the position of the sink node. Each node already knows the position of sink node and neighbor nodes from interest propagation. This directional information can be used to find closer nodes to the sink among neighbor nodes by comparing with the reference distance (D_ref_) of the sender S. The distance to the sink node of each node was considered to avoid the loop and longer hops caused by selecting an irrelevant node with inverse direction.

(2) $$Dk=\sqrt{{\left(xs-xk\right)}^{2}+{\left(ys-yk\right)}^{2}}$$

Equation (2) is from Pythagorean Theorem to calculate the distance between two nodes. Each node is aware of the position of neighbors through the interest propagation. For example, the distance *D_k_* to a sink at node *k* (Sink: *x_s_, y_s_* and N_k_: *x_k_, y_k_*) can be calculated from the Equation (2). When the sender S finds the closest node among neighbors, the distance is calculated among neighbors to compare with the reference distance.

The node selection process of the proposed algorithm is as follows. When a sender S receives the packets of both interest and data from the neighboring nodes, it calculates the air time cost for each node and updates it into its neighbor table from Equation (1). After that, the nodes that are closer than the reference node (sender, S) were selected as the candidate nodes. Finally, RA chooses a target node with the highest air time cost among candidate nodes.

**Figure 2.**
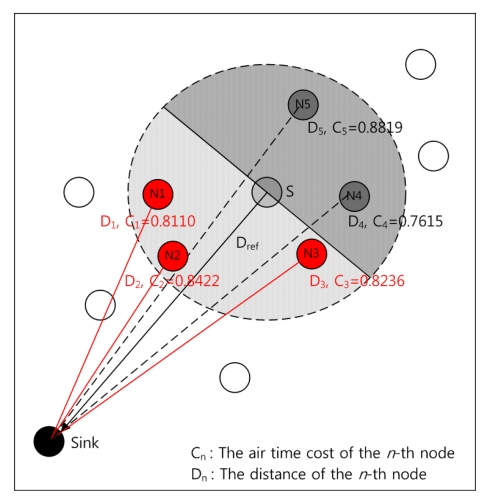
The node selection scheme of the proposed algorithm.

Figure 2 depicts the node selection process for the proposed algorithm. In the figure, N_1_, N_2_, N_3_ are selected as candidate nodes. Among them, N_1_ will be selected as the next node because it has the smallest value of the air time cost. Although the node N_4_ has the best air time cost (0.7615) among the neighbors, N_4_ will not be selected as the candidate because the direction to N_4_ is inverse to the sink node from the sender S.

The reason for selecting N_1_ instead of N_4_ with the best air time cost is as follows. Firstly, in order to prevent a sender selecting long route or loop, RA chooses candidate nodes with shorter distance than the reference distance of the sender. Secondly, RA chooses an optimum target node among candidate nodes with highest link quality which can reflect on the current network's status.

#### 3.2.4. The fast re-route scheme

In the proposed algorithm, the route was determined by the air time cost, but lossy situation still occurs when a next target node fails. At the moment, even the proposed algorithm cannot send data to the failed node without the fast re-route scheme, because the sender S has not updated the route failure information if the node error occurs at unspecified time. This route failure problem can be promptly fetched by using the fast re-route scheme. When a node detects the route failure, the route failure flag was set to 1. Then, the fast re-route scheme promptly starts to select alternative route that will replace the failed route.

The route failure flag is set by the RTS/CTS mechanism of the 802.11b MAC CSMA/CA protocol [28]. The RTS/CTS is a probing process before sending data to avoid interference among neighbors and hidden terminal problem. Since the utilization of RTS (Request To Send) and CTS (Clear To Send) packets gives advantages to the cross-layer design, the RTS/CTS mechanism is used to detect route failure in RA.

**Figure 3.**
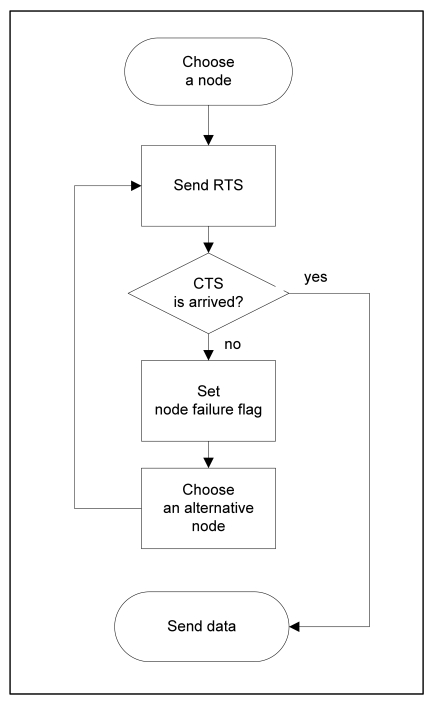
The flow chart of fast re-route scheme.

Figure 3 shows the process of the fast re-route scheme of RA. The sender S selects a target node N to forward sensing data based on RA. Before S forwards the data, S broadcasts the RTS packet to neighbors to check a route for making a connection between S and N. If N receives the RTS from S successfully, N sends the CTS to S as a response to the RTS. At that time, S checks the recipient of CTS from N until the retransmission of RTS exceeds the retry limit. If S does not receive the CTS from N, S updates the route failure flag of N in its neighbor table. After that, S starts the algorithm to determine an alternative node instead of N to re-route packets.

In this case, the route failure flag is included to decide an alternative node in addition to the air time cost and direction. The failure node, N was removed from the list of candidate nodes, and S chooses another node which has the best air time cost among remained candidate nodes in the table. After S selects a new target node, S starts to run the fast re-route scheme repeatedly until the data transmission succeeds.

#### 3.2.5. Link asymmetry of wireless network

RA showed better performance than DD in lossy WSNs, but link asymmetry still existed in RA. The link asymmetry is a well known problem which may affect variation of upper layer in wireless networks [29]. Especially, link asymmetry has a greater impact on the routing layer than on MAC layer.

In [29] the authors proposed solutions to cope with the asymmetric problem on wireless links at different layer: multi-round discovery, learning function, etc. A multi-round discovery technique needs multiple rounds of flooding to explore different paths with a large overhead. A learning function can be done with the help of RTS/CTS in the 802.11(DCF) MAC layer. This learning function allows a node to remember such an asymmetric link through verification of ACK to the response of CTS.

However, DD just delegates a solution for addressing the link asymmetry to MAC layer. In contrast, RA adopted two available solutions among the proposed methods to overcome the asymmetric problem at different layer. A multi-round discovery was operated at network layer and the learning function was run at MAC layer. Updating the air time cost is a multi-round discovery technique at network layer in RA, whenever a node receives both the interest and data packet. This method reduces high overhead of DD due to the multiple round of flooding.

In addition to the multi-round update of the air time cost, RA adopted a fast re-route scheme to guarantee the symmetry communication at MAC layer. If a node had lossy situation on a certain link, the fast re-route scheme was able to detect the link asymmetry. After the fast re-route scheme updated such asymmetric information on that link, the failed link was deleted from the neighbor table for an alternative route decision.

As a result, RA is able to guarantee a symmetric link to transmit data across the selected route using the multi-round update of the air time cost and the fast re-route scheme as an active method. RA can also provide optimum selection of a route without additional overhead and complexity. When the WSNs became unstable, these additional features of RA showed more improvement than DD.

## 4. Performance Analysis

### 4.1. Definition of the Error Model

Generally, the route of DD is established along the shortest path between source and sink. As the time passes, the energy of a node can be drained very quickly on the shortest path. The rapid dissipation of a node's energy brings node failure resulting in lossy situation. Consequently, a routing algorithm extends the shortest path including adjacent neighbor nodes instead of failed nodes to keep the connectivity of WSNs. We defined this extended path including the shortest path as a Main Communication Path (MCP). The MCP is an extended path constructed by a group of nodes enclosing the shortest path and adjacent neighbors of the path.

DD is designed to transmit most data over the MCP consisting of the shortest distances between nodes. As referred from [12,23], the behavior of the data transmission through the MCP is the main communication characteristics of DD. Although we can select an error node irrespective of the MCP, if we select error nodes with this method, the selected node does not participate in the transmission as a forwarding node. For this reason, the selection of the error nodes located on the MCP can emulate lossy situation better than that of uniform random selection regardless of MCP to clarify the problems of lossy WSNs.

For example, in the case of one source and one sink at each corner (left-bottom and right-top), the shortest path extended to adjacent neighbors forming a diagonal shape.

**Figure 4.**
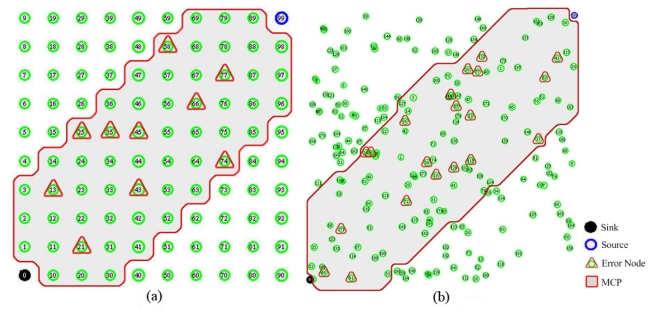
The topology used in the simulation (i.e., error rate: 10%). **(a)** Grid topology, **(b)** Random topology.

Figure 4 shows MCP used for each topology in the simulation. In Figure 4, a gray area indicates MCP and red triangle represents error-prone node with a given error rate (i.e., 10%) for each topology. The error group stands for the potential error nodes in the MCP. The error rate indicates the number of selected nodes in all nodes of topology, but the error-prone node should be randomly selected from MCP. The error rate represents the instability of lossy WSNs at a given error rates (%).

For example, the error group on the MCP is composed of 56 nodes for the grid topology and 112 nodes for the random topology. If the error rate is 5%, five nodes are selected as error-prone nodes from the nodes located on MCP at every selection. That is, in each selection, error nodes will be randomly selected from the sensor group on the MCP according to given error rate.

To model lossy WSNs, we make the randomly selected nodes to be blinked for a random time. Blinking selected nodes emulate the error-prone nodes to generate lossy situation as a random failure. The random failure of the selected error nodes is to avoid influence on interest propagation period for setting up gradient in DD. The interest propagation period is important to recover the lossy situation in DD. However, the lossy situation may occur in lossy WSNs independent with the interest propagation period as random failure. The author of DD mentioned the interest propagation period is a protocol design factor to trade-off between overhead and robustness.

The interest propagation period on time of original DD is a sum of fixed interval (30 seconds) and random time with large variance (range: a few ∼ hundreds seconds). However, this randomness might affect the number of resulted packets and the small interest interval of DD made a lot of interests flooding which might be a burden to analyze the effect of lossy WSNs.

Since the main purpose of lossy WSNs model is to generate unpredictable errors independent with the interest propagation period, we eliminates the randomness of DD to avoid difficulties in analyzing the result. Instead, in order to make the unexpected lossy situation, the fixed interval (60 seconds) is used for interest propagation and randomly selected nodes blinked for random time on MCP during the simulation.

### 4.2. Simulation Environment

For the performance analysis of the reliability in lossy WSNs, we measured the data deliver rate, the protocol overhead, and the average transmission delay time during simulations with *ns*-2 simulator [30]. We performed five simulations to investigate the diverse effect of lossy WSNs under different network conditions. In the first simulation, one source and one sink are deployed to compare grid topology with random topology. The second simulation is performed to analyze the difference between the different position of a sink and multiple sources as compared with the first simulation.

In the first simulation, 100 sensor nodes are located in the 200 m × 200 m network topology for the grid topology. 200 sensor nodes are deployed in the same network size as a uniform random distribution for a random topology. A sink and a source are placed at each corner during the first simulation.

For the analysis with multi sources and one sink, the network topology is the same as the grid topology, but the number of sources and a sink position is different. The shape of MCP depends on the position of sources and sinks. There are three different types of MCP in multi source experiments.

DD implemented the 802.11b MAC model to emulate realistic sensor radio [12] in the *ns*-2. We followed the same radio model and energy model used in DD. The transmission range of a node was only changed to 30 m to maintain the macroscopic connectivity of a sensor field. Other parameters were not modified to exclude any influence on the original DD. The detailed simulation parameters are shown in Table 2.

### 4.3. The Performance Metrics

The authors of [31] summarized the performance metrics to aid developing realistic sensor network models. Especially, the energy efficiency, fault-tolerance, and latency should be considered to evaluate sensor network protocol. However, we intended to mainly concentrate on fault-tolerance and latency according to the increment of instability in WSNs. These metrics can provide evidence for the robustness of a routing protocol in lossy WSNs. A fault-tolerance can be represented by means of a data delivery rate and protocol overhead. An average transmission delay indicates the one-way latency between transmitting data and receiving it at a sink.

We measured the data delivery rate, the protocol overhead, and the average transmission delay to compare One Phase Pull (OPP) with the proposed algorithm (RA) in lossy WSN model with various topologies:

(3) $$\text{Rdelivery}\left(\%\right)=\frac{\text{Nsink}}{\text{Nsource}}\times 100$$

Data delivery rate (*R_delivery_*) is the percentage of successfully delivered packets at a sink, where *N_source_* is the number of packets sent from the source node and *N_sink_* is the number of successfully received data at a sink node in Equation (3):

(4) $$\text{Oprotocol}=\frac{\text{Ncontrol}}{\text{Ndata}}$$

Protocol overhead (*O_protocol_*) is a ratio between the number control packet and data packet, where *N_cotrol_* is the number of control packets and *N_data_* is the number of data packets in Equation (4). A large *O_protocol_* indicates that many control packets is needed to deliver data to a sink successfully.

(5) $$\text{Tavg}=\frac{\sum \left(\text{Tsink}-\text{Tsource}\right)}{\text{Ndelivery}}$$

Average transmission delay (*T_avg_*) represents the average time difference on successfully received data at a sink from the source node, where *T_source_* is the sending time of data at source and *T_sink_* is the receiving time of the same data at a sink and *N_delivery_* is the number of delivered data in Equation (5).

## 5. Performance Analysis

### 5.1. Grid Topology

**Figure 5.**
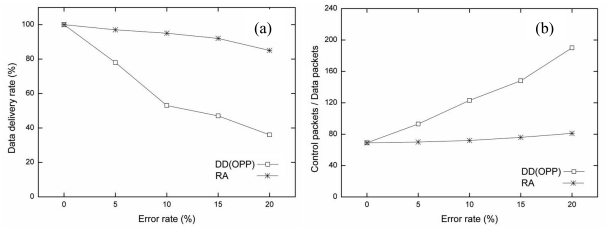

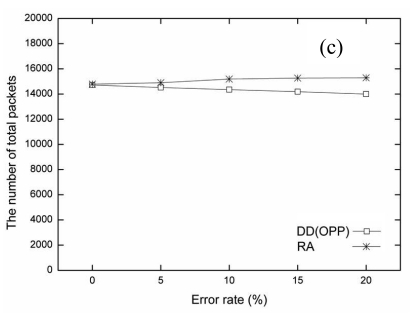
Performance comparison based on error rate in grid topology (0%, 5%, 10%, 15%, 20%). (a) Data delivery rate, (b) Protocol overhead, (c) Total number of packets.

In the first simulation, the performance metrics of original DD (OPP) and the proposed RA were compared in a grid topology with error rates of 0%, 5%, 10%, 15%, and 20%. Figure 5 shows the simulation results in the grid topology. From Figure 5(a), as the error rate increases, the data delivery rate decreases in both algorithms. Both OPP and RA show 100% delivery for the stable condition (error rate, 0%). However, when the error rate increases to 20%, the data delivery rate of RA is four times better than OPP. The reason is RA used the air time cost and route failure information for the current wireless link. That is, RA can adjust in the lossy situation.

The protocol overhead of RA is also smaller than OPP. Figures 5(b) and 5(c) show the simulation results. The ratio of control packets over data packets is shown in Figure 5(b) and the total number of packets is shown in Figure 5(b). As the error rate increases in OPP, the number of control packets increases rapidly in inverse proportion to the number of data packets. If the route is broken during data transmission period, the data can be sent back to the sender. When the sender receives the packet from the receiver node again, the sender assumes the route is a loop. Removal of the packet loop relies on negative reinforcement.

The protocol overhead of OPP increased more as the number of control packets and transmission failures increased. RA maintains low protocol overhead compared to OPP, RA does not affect the error rate significantly in spite of increasing the error rate with RA. RA keeps a higher data delivery rate at every error rate. In addition, the air time cost and the fast re-route scheme are used to provide reliable communication with RA. Additional control packets do not need to be sent to remove the loop. RA re-routes to an alternative path after a failure. For this reason, although the total number of packets with RA is larger than that with OPP, the data delivery rate and the protocol overhead of RA are better than OPP. Table 3 summarizes the results of the simulations.

**Figure 6.**
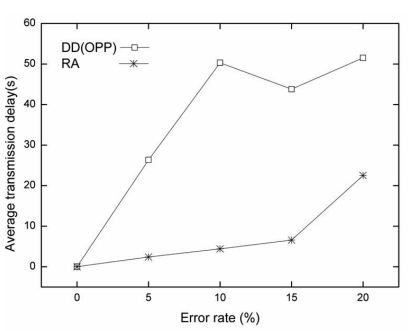
The average transmission delay for each error rate in grid topology (0%, 5%, 10%, 15%, 20%).

Figure 6 describes the average transmission delay for each error rate. The average transmission delay was measured by successfully delivered data from a source to a sink as the time elapsed during simulation. When the data matches the interest at a source node, the source node sends data to a sink node at every five seconds.

The average transmission delay of RA is quite smaller than that of OPP for all error rates. As the error rate increases in lossy WSNs, OPP suffers from a data loss. In this case, OPP cannot send data to a sink unless the route is set up again by interest propagation. When data transmission fails, the maximum delay time follows the interest propagation cycle. In this paper, we used 60 seconds as the interest propagation cycle. Since RA is able to re-route using the air time cost and route failure information during data transmission period, RA shows a small average transmission delay. However, if the error rate is above 15%, the alternative routes decrease, and the average transmission delay increases slightly. The results are shown in Figure 6.

### 5.2. Random Topology

In the second simulation, the performance metrics of the original DD (OPP) and the proposed RA were compared in a random topology with diverse error rate at 0%, 5%, 10%, 15%, and 20%.

**Figure 7.**
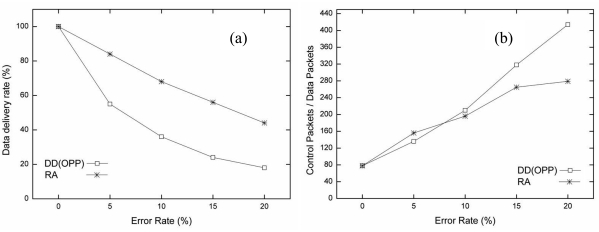

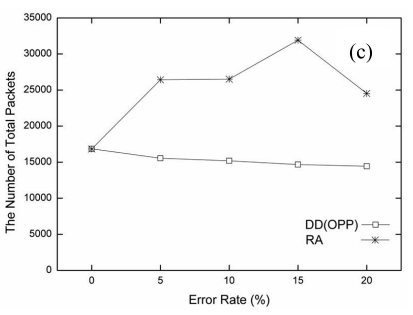
Performance comparison for each error rate in random topology (0%, 5%, 10%, 15%, 20%). (a) Packet delivery rate, (b) Protocol overhead, (c) Total number of packets.

Figure 7 shows the data delivery rate, the protocol overhead and the total number of packets for each error rate with the random topology. Compared to the grid topology, the data delivery rate of OPP decreased more quickly with random topology as the error rate increased. From our analysis, the data delivery rate tends to be affected by errors due to the irregular distance among neighbor nodes. Because the irregular distance leaves few candidate routes in addition to the failure node, the number of alternative routes also decreases. As a result, the data delivery rate of OPP is very low with the random topology. In the random topology, RA shows a poor data deliver rate compared to the grid topology. However, RA shows still better data delivery rate than OPP as shown in Figure 7(a).

The protocol overhead of both OPP and RA with random topology is four times less than that with the grid topology. Since the number of nodes increases and the distance among neighbor nodes gets random, the number of control packets also increases. The number of data packets in the network decreases with increasing error rate, as shown in Figures 7(b) and 7(c). Furthermore, the number of control packets with the random topology increases with an increasing number of nodes, because the distance among candidate nodes are different.

In the random topology, the total number of packets was increased to three times more than that with the grid topology. As the total number of packets increases and the data delivery rate decreases, the protocol overhead increases consequently. RA shows a higher total number of packets than OPP in Figure 7(b). This results from the re-transmission of packets that provide reliable communication through the re-route scheme. Nevertheless, the protocol overhead of RA shows better performance as the error rate increases. This proves that proposed RA maintains reliable communication and does not affect the data delivery rate regardless of error rates or topologies. Table 4 shows all of the metrics in the simulation results.

**Figure 8.**
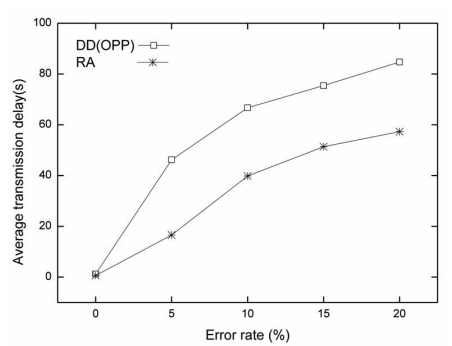
The average transmission delay for each error rate in random topology (5%, 10%, 15%, 20%).

Figure 8 shows the average transmission delay time for each error rate in a comparison between OPP and RA. The average transmission delay of RA is also smaller than that of OPP for all error rates with the random topology. Compared with the grid topology, as time increases, OPP and RA show more delay at a certain time, as shown in Figure 8. In particular, the maximum average transmission delay with OPP is seen more frequently over all error rates. The lossy situation occurs more frequently with random topology due to its random characteristics. The average transmission delay of RA also exceeds that of the grid topology. However, compared to OPP, RA shows small average transmission delay with the random topology.

In the random topology, less data is successfully sent to the sink node compared to grid topology. The average transmission delay occurs, because the distance between neighbor nodes is different from a random deployment of nodes. This random distance tends to affect the gradient set up for each route when an error occurs at a node. As a result, OPP shows a higher average transmission delay at every error rate compared to OPP in the grid topology.

RA has a smaller average transmission delay for data transmission than OPP. However, compared to the grid topology, the random distance affects the setup of the route in RA as well. Although the distance was considered using RA algorithm, this case is slightly different to the effect of random distance. If a target node is out of range from the sender, the node cannot be a candidate node in the sender's alternate route. In this algorithm, candidate nodes within the communication range of the sender node are only considered as alternate routes, accounting for the low performance of random topology compared to grid topology.

### 5.3. Multiple Sources with Different Sink Position

This section describes the effects with different position of multiple sources and a single sink for further analysis. The error group may vary according to the position of sources and a sink. The different number of sources and the position of a sink may slightly degrade the advantages of proposed RA due to the different shape of error group in MCP.

**Figure 9.**
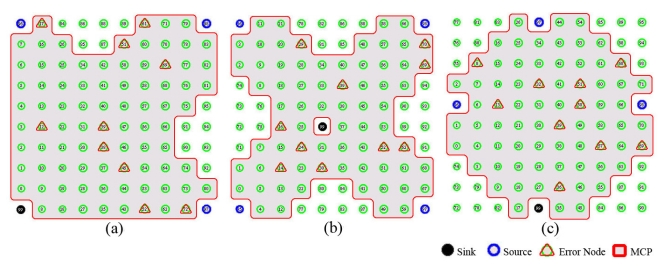
The topology used in the simulation for different MCP including multiple sources with different sink position (i.e., error rate: 10%) **(a)** Arrow type, **(b)** Cross (X) type, **(c)** Diamond type.

In this experiment, we performed three simulations with different MCP including multiple sources and single sink. Figure 9 shows the deployment of different position of nodes and MCP. Basically, when the path between a source and a sink becomes longer, the lossy situation tends to frequently occur. For the simulation, multiple sources are placed at the edge of each topology with a different sink positions to maximize the effect of the lossy situation. Figure 9 shows each topology and the node deployment which defines the error group of MCP.

We defined three topology based on the shape of each MCP to identify topologies: arrow type, cross type, and diamond type. Figure 9(a) shows the node deployment of arrow type that includes three sources and one sink placed at each corner. Figure 9(b) illustrates the topology of cross type which places four sources at each corner and one sink at the center. The third topology that includes three sources and one sink at the center of four each end edge as shown in Figure 9(b).

**Figure 10.**
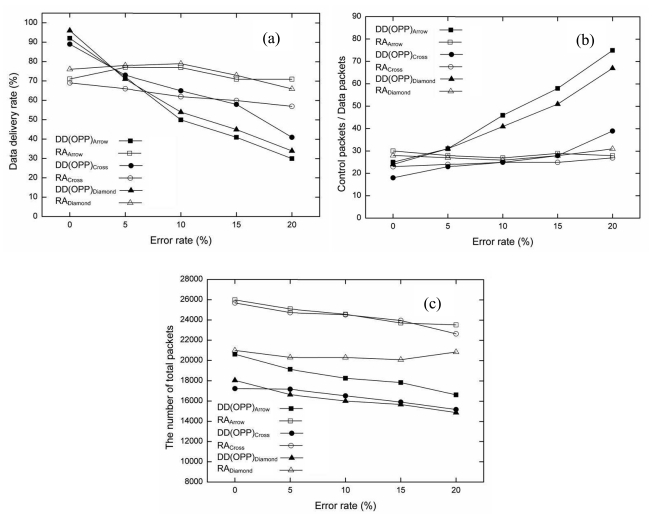
Performance comparison based on error rate in different topologies with multiple sources and different sink position (0%, 5%, 10%, 15%, 20%) (a) Packet delivery rate, (b) Protocol overhead, (c) Total number of packets.

Figure 10 shows the performance comparison in different MCP with the different position of multiple sources and a sink. According to our analysis, the arrow type of MCP shows the best performance. The second is the diamond type and the third is the cross type in order. In the Figure 10(a), the data delivery rate of both RA and OPP decreased smoothly as compared with the first simulation (one source and one sink). The increment of the number of source and different position of a sink influence the delivery rate of both algorithms. The topology with the arrow type of MCP showed the best delivery rate than others.

However, when the network is stable (error rate: 0%), all of OPP show better data delivery rates (almost 95%) than the case of RA (around 70%). The performance degradation of RA is resulted from interferences increased around a sink. When data arrives at the neighbors of a sink, a neighbor has to choose a next hop to send data to a sink. At the same time, if another neighbor tries to choose a route as the same way, the re-route scheme of the node is executed to avoid lossy situation. The packets generated by the re-route scheme may be additional overhead. For this reason, if a sink has insufficient candidate nodes, the interference may increase more in RA.

Figure 10(b) shows the comparison of protocol overhead for each topology. All of OPP show larger protocol overhead than RA. Especially, when a sink is located at the corner (left-bottom), the interest flooding increased to recover lossy situation. Since the longer distance between a source and a sink enables the error probability to be high, protocol overhead is usually quite large in two topologies (arrow type and diamond type).

The comparison of total number of packet is as shown in Figure 10(b). All of OPP show a large number of total packets resulting from generating many control packets in the algorithm. Oppositely, the number of total packets of RA is smaller than OPP. Compared to the first experiment (one source and one sink), the number of total packets of RA increased three times more due to the multiple sources. Although the total packets increased in OPP, OPP shows disadvantages with multiple sources and a different sink position. In contrast, RA shows big difference of the total packets of DD regardless of the different MCP.

**Figure 11.**
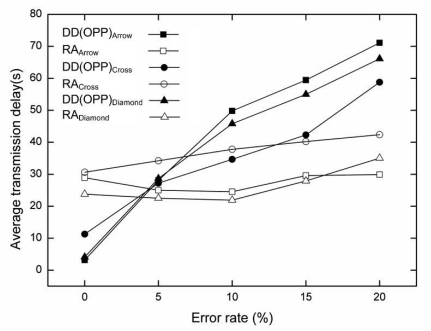
The average transmission delay for each error rate with multiple sources and different sink position (0%, 5%, 10%, 15%, 20%).

Figure 11 describes the average transmission delay of the each topology. The average transmission delay of RA is smaller than that of OPP for all cases except the topology with cross type of MCP [see Figure 10(b)]. As the error rate increases, the average transmission delay of OPP increases rapidly compared to that of RA. RA shows small difference of average transmission delay even at higher error rate. When the error rate is less than 5%, OPP has small average transmission delay than the case of RA at all types of MCP. However, the average transmission delay of RA slightly increases as the error rate increases. In contrast, the delay of DD increases rapidly according to the increment of the error rate. The result shows that DD is greatly influenced by the instability of the lossy situation.

The average transmission delay of the cross type is worst than others as well as other metrics, because the four sources may use different path. Compared with other type, a cross type intermediate node has a different path that creates additional overhead from maintaining multiple paths for each source node. In other types of topology, the path from a source to a sink can be shared by an intermediate node. This behavior of the cross type to maintain multiple paths can be a reason for degrading performance.

We verified that RA shows even better performance than DD with different position of multiple sources and one sink. DD suffers from lossy situation a lot more than RA in this environment. DD remains the problem which cannot provide reliable and efficient communication in lossy WSNs. However, RA shows performance improvement regardless of topology, different position and multiple sources. The performance degradation of RA can be seen as little at the experiment with different MCP, but the performance fluctuation according to the error rate is less than the first experiment with one source and one sink.

## 6. Conclusions

WSNs have unique characteristics such as resource limitations, communication flow from many to one, and deployment in error prone environments. Many routing protocols have been developed to address these characteristics. These routing protocols mainly contributed to prolonging network survivality by saving energy during communication, but most research studies do not provide reliable communication in an error-prone environment. Lossy WSNs results from contention and interference among nodes, node mobility, fading by object, or node failure from battery discharge and physical damage.

We proposed a radio-aware routing algorithm to improve the reliability of DD in lossy WSNs. DD is well-suited for WSNs' routing because it uses local interactions among distributed nodes. DD also provides data-centric, robust, and scalable models, but previous research did not consider the unstable network. The data delivery rate of DD decreased very quickly in an error prone environment.

In this paper, to handle the lossy environment of WSNs properly, RA algorithm which uses radio information was proposed. RA algorithm is a data centric routing protocol like the original DD, but RA algorithm can be aware of the network's status by adopting a cross-layer approach between MAC and network layers. Furthermore, a fast re-route scheme was also applied to provide consistent and quick re-transmission for reliability. The radio-aware reliable routing algorithm contributed to providing QoS, reliability, and reduction of DD's overheads for lossy WSNs including scalability and interoperability.

To prove the effectiveness of the proposed algorithm, the simulation was performed with *ns*-2 under various conditions. Lossy WSNs were emulated by randomly blinking sensor nodes to create random node failures. According to the simulation results, the radio-aware routing algorithm showed better performance for each error rate. The error model for five simulations contained grid and random topologies with diverse MCP including multiple sources and different sink position at error rates of 0%, 5%, 10%, 15%, and 20%. As the error rate increases, the proposed solution showed higher data delivery rate, lower protocol overhead, and lower average transmission delay as compared to DD regardless of the error rates and topologies.

The simulation results demonstrate the possibility of providing a reliable transmission method for QoS requests in lossy WSNs by using radio-awareness. However, when a node chooses its forwarding node based on our algorithm, the re-route scheme consumes more energy due to packet retransmission. The energy consideration and mobility will be addressed in future work.

## Figures and Tables

**Table 1.** Neighbor table.

Node id	Position x	Position y	Air time cost	Route failure flag
22	50	50	0.08819	1
**23**	**50**	**70**	**0.09115**	**0**
24	50	90	0.09115	0
⋮	⋮	⋮	⋮	⋮
32	70	50	0.07311	1
44	90	90	∞	0

**Table 2.** The simulation parameters.

Network size	200 m × 200 m
Topology	Grid, Random
The number of sink	1
The number of source	1, 3, 4
Number of nodes	100 (Grid), 200 (Random)
Transmission range	30 m
Initial node energy	30 J
Location of sink node	(10, 10)
Location of source node	(190, 190)
Error rate	5%, 10%, 15%, 20%
Simulation time	1,000 s
Interest duration	60 s

**Table 3.** Simulation result with grid topology.

**Error rate (%)**	**Delivery rate**		**Overhead**		**Total packets**	

	**OPP(%)**	**RA(%)**	**OPP**	**RA**	**OPP**	**RA**

No error	100	100	69	69	14,715	14,787
5	78	97	93	70	14,516	14,897
10	53	95	123	72	14,352	15,187
15	47	92	148	76	14,184	15,262
20	36	85	190	81	13,995	15,286

**Table 4.** Simulation results with random topology.

Error rate (%)	Delivery rate		Overhead		Total packets	

	OPP(%)	RA(%)	OPP	RA	OPP	RA

No error	100	100	274	274	49,481	49,908
5	55	98	489	277	48,818	50,218
10	34	95	810	290	48,992	51,045
15	24	89	1174	301	48,993	50,059
20	16	80	1726	346	48,996	51,954
